# The Role of Nutrition in HIV-Associated Neurocognitive Disorders: Mechanisms, Risks, and Interventions

**DOI:** 10.3390/life15060982

**Published:** 2025-06-19

**Authors:** Carlotta Siddi, Jihane Balla, Christy Agbey, Paola Fadda, Simona Dedoni

**Affiliations:** 1 Department of Biomedical Sciences, Division of Neuroscience and Clinical Pharmacology, University of Cagliari, 09042 Cagliari, Italy; c.siddi2@studenti.unica.it (C.S.); jihane.balla@unica.it (J.B.); paola.fadda@unica.it (P.F.); 2 Department of Neuroscience, Georgetown University Medical Center, Washington, DC 20057, USA; cja72@georgetown.edu; 3 Neuroscience Institute, National Research Council of Italy (CNR), 09142 Cagliari, Italy

**Keywords:** human immunodeficiency virus (HIV), HIV-associated neurocognitive disorders (HANDs), anti-retroviral therapy (ART), acquired immunodeficiency syndrome (AIDS), food, body weight, inflammation

## Abstract

HIV-associated neurocognitive disorders (HANDs) refer to a range of cognitive deficits that afflict people living with the Human Immunodeficiency Virus (HIV). The fundamental processes of HAND include persistent inflammation, immunological activation, and direct viral impact on the central nervous system. Emerging research shows that nutritional status, especially food consumption and body weight, is critical in determining the course and severity of HAND. Malnutrition exacerbates neurocognitive impairment by increasing inflammation and oxidative stress, while obesity may contribute to HAND through the promotion of metabolic disruption, gut microbiota alterations, and systemic inflammation. Additionally, the introduction of antiretroviral treatment (ART) has substantially enhanced the prognosis of people living with HIV by lowering viral load and improving immune function. However, depending on the regimen, ART can cause changes in body weight, which may influence the progression of HAND. This emphasizes the intricate interplay between HIV, nutrition, body weight, and neurocognitive health. As a result, various dietary approaches are currently being investigated to improve the quality of life of individuals with HIV and possibly help prevent neurocognitive decline in this population. This review aims to elucidate the relationship between nutrition and neurocognitive function in individuals living with HIV, shedding light on aspects of HANDs related to diet, body weight fluctuations, and metabolic syndrome. It explores the shift from current pharmacological treatments to innovative non-pharmacological interventions, including specific dietary strategies, to support overall health and cognitive well being in HIV-positive people.

## 1. Introduction

The Human Immunodeficiency Virus (HIV) was first identified in the early 1980s, marking the beginning of one of the most significant global health challenges of modern times [1,2,3,4]. Subsequent research revealed that HIV targets the immune system, primarily CD4+ T cells, causing a gradual reduction in immunological function. HIV infection progresses through three main stages. The earliest phase, HIV acute infection, occurs 2 to 4 weeks after infection. Individuals may experience flu-like symptoms such as fever, headache, and rash. HIV rapidly multiplies, attacking CD4 cells of the immune system. The second phase is chronic HIV infection, also known as asymptomatic HIV or clinical latency. During this stage, HIV continues to replicate at low levels. This stage can last for 10 years or more and may eventually progress to acquired immunodeficiency syndrome (AIDS), which is the final and most severe stage. By this point, the immune system is heavily damaged, leaving the body vulnerable to opportunistic infections and malignancies [5]. HIV primarily spreads through unprotected sexual contact, blood transfusions, sharing needles, and from mother to child during childbirth or breastfeeding. High-risk behaviors, such as having multiple sexual partners and substance abuse, increase the likelihood of transmission. HIV incidence and prevalence have changed dramatically over the past four decades. By the end of 2023, approximately 39.9 million people globally were living with HIV [6]. Sub-Saharan Africa remains the epicenter of the epidemic, accounting for roughly 70% of global cases, with women and girls representing 44% of new infections. Despite a 60% reduction in new infections since 1995, there were still 1.3 million new HIV cases and 630,000 HIV-related deaths in 2023. This remains far from the global target, which aims to reduce HIV-related deaths to fewer than 250,000 by 2025 [6].

The introduction of antiretroviral therapy (ART) in the mid-1990s revolutionized HIV treatment. ART successfully reduces viral replication, halts the progression to AIDS, and allows people with HIV to live healthier and longer lives. ART involves the use of a combination of antiretroviral medicines that target distinct phases of the viral life cycle [7,8]. When taken consistently, ART can prevent the transmission of HIV [9]. Despite the success of ART, issues persist, including medication resistance, side effects, and the necessity for lifetime adherence. It is now widely recognized that individuals living with HIV are at a higher risk of developing metabolic and/or cardiovascular complications due to the effects of HIV itself, ART, and chronic inflammation. This increased risk can lead to a higher likelihood of conditions such as obesity, insulin resistance, dyslipidemia, and hypertension [10], which are key components of metabolic syndrome (MetS).

Another long-term complication of HIV, even in the era of effective ART, is the development of HIV-associated neurocognitive disorders (HANDs) [11]. The progression of these disorders is influenced by factors including persistent immune activation, viral reservoirs in the central nervous system (CNS), and aging. Neuropathological studies in patients have identified synaptic loss, neuroinflammation, and neuronal injury as key contributors. While ART reduces the incidence of severe HAND, mild and moderate impairments continue to affect quality of life, underscoring the need for targeted neuroprotective interventions. HAND encompasses a spectrum of neurocognitive impairments and is believed to result from chronic inflammation and immune activation within the central nervous system (CNS), driven by ongoing HIV replication, even in the presence of ART [12,13,14,15,16]. The prevalence of HAND varies, with estimates suggesting that up to 50% of individuals with HIV may experience some degree of neurocognitive impairment [11,12]. The onset of HAND can occur at any stage of HIV infection, but it is more common in individuals with advanced disease or those who have been living with HIV for a longer time [17,18]. Early identification and management of HAND are critical, because neurocognitive deficits can significantly impact an individual’s quality of life and ability to adhere to ART [14,19]. With effective ART, the life expectancy of people living with HIV has increased exponentially, approaching that of the general population in many cases [20]. However, the presence of HAND and other comorbidities can complicate long-term outcomes. Alongside medical treatments, dietary strategies are becoming an essential part of managing the health of individuals living with HIV and HAND.

Nutrition plays a vital role in supporting immune function, managing inflammation, and enhancing the body’s ability to cope with the effects of HIV and neurological impairments. Diets rich in antioxidants, vitamins (such as B vitamins and vitamin D), healthy fats (omega-3 fatty acids), and anti-inflammatory foods have been shown to improve both physical and cognitive health. Moreover, addressing specific nutritional deficiencies, such as low vitamin B12 or folate, which are common in people with HIV, can help alleviate symptoms of HAND. Malnutrition and significant weight loss, common in people with untreated HIV, are associated with faster disease progression and an increased risk of HAND [21]. Conversely, ART has been linked to weight gain in some individuals, which may also influence the risk of HAND through metabolic pathways [22,23,24]. Adequate nutrition is essential for maintaining a healthy weight, supporting immune function, and mitigating the side effects of ART. Integrating nutritional support into HIV care is therefore essential for optimizing treatment outcomes and enhancing the overall quality of life for people living with HIV [25,26]. Emerging research is also exploring the gut–brain axis and how microbiome health may influence both HIV progression and neurological health.

**Figure 1:**
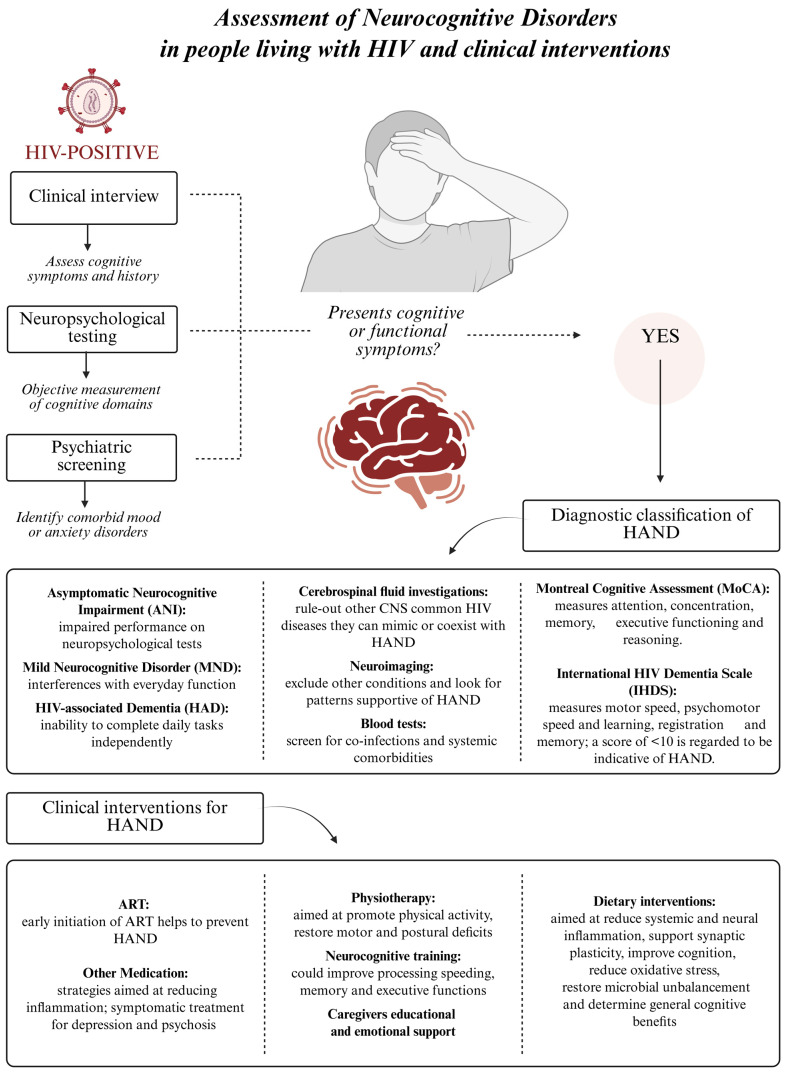
Flow chart describing the assessment of neurocognitive disorders in people living with HIV. The flowchart illustrates the assessment and clinical management of HIV-associated neurocognitive disorders (HANDs) in people living with HIV. The chart outlines the diagnostic assessment, including screening, neuropsychological evaluation, and classification of HAND subtypes, followed by clinical interventions aimed to ameliorate life expectancy and the quality of life of HIV-positive people.

Therefore, understanding the connection between diet, immune system function, and cognitive health is vital for developing comprehensive strategies that improve the long-term health and quality of life of people with HIV. While great success has been achieved in the battle against HIV, continued hurdles exist, particularly in treating long-term consequences of infection, such as HAND. This review will focus on understanding the complex interactions among MetS, HAND, and the role of nutrition (as illustrated in Figure 1) as a vital component in developing comprehensive treatment strategies to improve the lives of people living with these conditions.

## 2. Metabolic Syndrome and Body Weight in People with HIV

In recent years, significant efforts have been made to investigate HIV-associated metabolic syndrome (MetS). This is due to the fact that, after the introduction of ART, treated patients began experiencing various metabolic issues, including insulin resistance, dyslipidemia, fat redistribution, and elevated blood pressure [10]. MetS includes risk factors that increase the risk of people with HIV developing heart disease, stroke, and type 2 diabetes. Although the exact role that HIV infection plays in the development of MetS is unknown, it is now widely accepted that immune system dysregulation is the primary component that underlies this condition. People with HIV experience dysfunctional immune metabolism that affects key metabolic pathways, including glycolysis, glutaminolysis, and oxidative phosphorylation, ultimately impacting immune cell function and metabolic health [27]. For instance, HIV infection leads to dysfunctional glucose metabolism in T cells and monocytes through immune activation, resulting in the overproduction of glycolytic metabolites by CD4+ T cells and promoting the survival of HIV-infected cells [28].

In addition to HIV, ART has been closely linked to MetS. ART employs a combination of anti-retroviral drugs aimed at lowering the viral load, improving quality of life, and reducing mortality and morbidity rates of people living with HIV. ART also prevents HIV transmission by impeding HIV replication in people with HIV [29]. The FDA-approved antiretroviral medications are categorized into eight pharmacological classes [30], based on the mechanism by which each medication interferes with the HIV life cycle. The eight classes of medications include Nucleoside-analog Reverse Transcriptase Inhibitors (NRTIs), Non-Nucleoside Reverse Transcriptase Inhibitors (NNRTIs), Fusion inhibitors (FIs), Protease inhibitors (PIs), Chemokine receptor 5 (CCR5) antagonists, Integrase inhibitors, Post attachment inhibitors, and Pharmacokinetic Enhancers (NCBIs). The World Health Organization (WHO) states that first-line ART for adults should consist of two NRTIs plus integrase inhibitors, while second-line ART typically includes PIs and two NRTIs [31]. How HIV or ART affects MetS is not yet fully understood, in part because these metabolic changes likely result from a combination of factors. For example, PIs are most strongly associated with an increased risk of MetS development. In a study by Dressman et al., PIs such as ritonavir, indinavir, and amprenavir were shown to promote atherosclerotic disease by upregulating CD-36-dependent cholesteryl ester and suppressing cis-9-retinoic acid and peroxisome proliferator-activated receptor gamma (PPAR-γ). This led to lower levels of high-density lipoprotein (HDL) and higher levels of circulating triglycerides, total cholesterol, and low-density lipoprotein (LDL) [32]. Another study investigating the adverse effects of anti-HIV drugs (D:A:D study) showed that people treated with various ART regimens exhibited different plasma lipid profiles. This study found an increased prevalence of dyslipidemia in patients receiving an NNRTI-containing regimen, a single PI-containing regimen, or a dual PI-containing regimen [33]. This may have substantial clinical implications, including increasing cardiovascular disease risk and worsening overall health outcomes. Moreover, it was recently discovered that the U.S. population has the highest risk of developing MetS, followed by Southeast Asia and the Western Pacific. The incidence of MetS is 1.5 times greater in patients receiving ART than in those without treatment [34]. In the GEMINI-1 and GEMINI-2 clinical studies, two different regimens of ART were found to cause comparable weight gain, although it may have a distinct effect on other risk factors as well. One regimen included Dolutegravir (DTG) combined with Lamivudine (3TC), while the other included Dolutegravir (DTG) combined with Tenofovir disoproxil fumarate (TDF) and Emtricitabine (FTC). Patients in the two-drug regimen (DTG/3TC) and those in the three-drug regimen (DTG/TDF/FTC) gained identical amounts of weight, but their lipid levels changed differently, with the DTG/3TC regimen showing a better lipid profile at 48 and 96 weeks [35]. Low baseline CD4 T-cell count and high baseline HIV-RNA levels were also associated with weight gain. Tenofovir alafenamide (TAF) and integrase strand transfer inhibitor (INSTI)-based regimens were recently identified as ART classes most strongly linked to weight gain [36,37].

Overall, the incidence of increased MetS in people with HIV has varied significantly across different studies (18–33%) and is not only influenced by the type and duration of ART, but also by factors such as gender and age [38,39]. For example, women with HIV experience a significantly greater increase in BMI after starting ART compared to men. In a study by Bares et al., data from three U.S.-based randomized controlled trials of participants initiating ART showed that, over 96 weeks, women gained an average of 1.91 kg/m^2^, compared to 1.39 kg/m^2^ in men. The BMI changes in women were greater than the average estimated differences at the beginning of the study [40]. In accordance with this, Chillo et al. (2024) conducted a prospective cohort study of 4000 people with HIV undergoing ART in Tanzania, and found that after one year of treatment, female subjects had a higher BMI compared to males, with a difference of 2.2 kg/m^2^ [41]. In this study, clinical factors such as lower CD4-T-cell counts, advanced HIV disease stage, and pulmonary tuberculosis infection were positively associated with greater weight gain. In addition, women living with HIV are more likely to develop obesity, insulin resistance, bone density decline, and dyslipidemia, while men are more prone to hepato-steatosis and hepatic fibrosis. These differences may be influenced by factors such as sex hormones, menopause, and chronic inflammation, which are greater in women living with HIV than in men [42]. Finally, ethnicity is another significant risk factor that could cause weight changes in people undergoing ART. For instance, it has been reported that black women undergoing ART gain twice as much weight as non-black women [22]. After starting ART, half of the participants gained at least 3% of their body weight over nearly two years of follow-up, with a median increase of 2.0 kg. These changes were linked to lower HDL levels and a higher total-cholesterol-to-HDL ratio, with no reported changes in LDL [22].

### 2.1. ART-Driven Dysbiosis and MetS

The impact of ART on MetS has also been investigated in the context of the gut microbiome composition. The changes in the architecture of lymphoid tissue after the depletion of gastrointestinal CD4+ T lymphocytes due to HIV infection [43] compromises the integrity and functionality of the gut mucosal barrier. This leads to peripheral inflammation and gut microbiota disbalance in people with HIV [44]. In support of this, studies have shown that patients with HIV often exhibit an imbalanced microbial composition, characterized by a shift toward pro-inflammatory species at the expense of beneficial bacteria [45]. Although HIV itself can impact gut microbiota composition and function, both HIV and ART potentially contribute to metabolic disturbances. It has been demonstrated that certain medications within ART regimens have significant dysbiotic effects on the diversity and composition of gut microbiota. The results from several studies investigating the gut microbiome composition in people with HIV are summarized in Table 1.

### 2.2. Metabolic Syndrome and Neurocognitive Impairments in People with HIV

In addition to its effect on the lipid profile, MetS is a widely recognized risk factor for the development of neurocognitive impairments (NCIs). In fact, MetS has been associated with various forms of dementia, including Alzheimer’s disease (AD) [49] and mild cognitive impairment (MCI) [50]. Risk factors related to metabolic imbalance, such as cardiovascular disease [51], diabetes [52], obesity, and dyslipidemia [53], could exacerbate the course of dementia, particularly AD, in a number of ways. For example, dyslipidemia may impair cognition by promoting neuroinflammation through microglial activation, disrupting blood–brain barrier integrity, and reducing cerebral blood flow, thereby depriving the brain of oxygen and nutrients. The link between MetS and NCIs has garnered growing interest, especially in cases demonstrating an elevated risk for cardiovascular disease and stroke (driven by MetS associate disease). Furthermore, other populations, including people with HIV who are more vulnerable to developing ‘’Salt-sensitive hypertension’’ [54], could be affected. This form of hypertension, driven by genetic modifications in the expression of the epithelial sodium channel (ENaC), may influence the preference for salty foods in people with HIV, thereby increasing their chance of developing vascular issues [55]. This may affect cognition by leading to white matter lesions, blood–brain barrier disruptions, oxidative stress, and neurodegeneration. Extensive studies examining the association between MetS and cognition in people with HIV conclude that obesity [56], diabetes, and insulin resistance [57,58] are associated with a higher prevalence of NCIs in people with HIV. Although the mechanisms by which MetS exacerbates NCI in people with HIV remain unclear, research has shown that cerebral artery damage, impaired brain-glucose metabolism, and altered blood–brain barrier permeability play important roles [59].

Additionally, people with HIV undergoing ART exhibit elevated cholesterol levels, which correlate positively with NCIs. The use of statins partially slowed the progression of the cognitive impairment [60] and reduced the number of circulating CD16+ monocytes, which have high pro-inflammatory properties in individuals with HIV [61]. Moreover, a 2017 study by Okafor et al. examined a total of 90 adults with HIV with a mean age of 46 years, 86.7% of whom were undergoing ART [62]. Participants underwent a BMI assessment, inflammatory cytokine profile analysis, and neurocognitive function evaluation. The results showed that obesity and IL-16 levels were significantly associated with slower processing speed. This suggests that a higher BMI, together with elevated systemic inflammation, may contribute to cognitive dysfunction among adults with HIV. In a study by Yu et al., 109 people living HIV with MetS underwent a neurocognitive examination involving a battery of tests and a psychiatric evaluation [63]. The results showed that metabolic abnormalities, particularly diabetes and higher levels of triglycerides, were strongly associated with NCIs, including impaired learning, motor skills, and executive function. Furthermore, when examining disparities in HIV-associated neurocognitive function between Hispanic and non-Hispanic people with HIV and MetS receiving ART, both groups showed an overall impairment in neurocognitive function [64]. Overall, these results highlight the need for the early identification of people with HIV who are at risk for developing MetS, and the implementation of preventive approaches, such as a controlled dietary regimen, to reduce the risk of developing NCIs. Further study is needed to delineate the exact mechanisms by which these risk factors affect cognition.

## 3. Altered Brain Metabolism and Neuronal Dysfunction Underlying Neurocognitive Impairments in People with HIV

**Figure 2:**
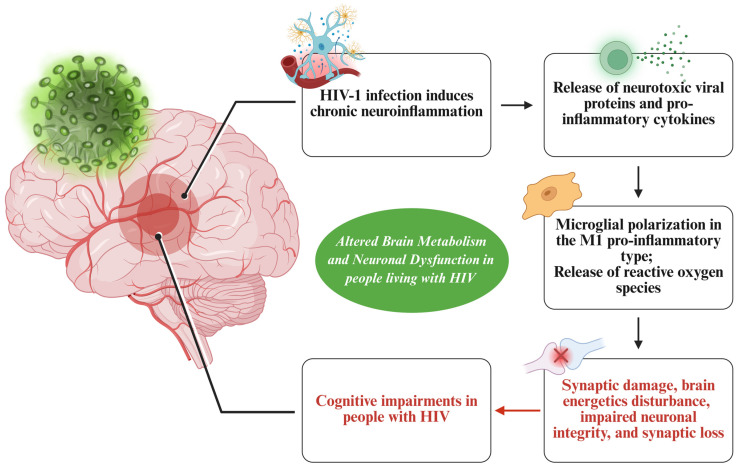
Cognitive impairments in people with HIV. This figure summarizes the mechanisms that occur in people with HIV and neurocognitive disorders. The virus itself can cause neuroinflammation, which is followed by the release of pro-inflammatory markers and microglial activation; the release of oxygen reactive species, leading to synaptic loss; and cognitive impairments.

As discussed, virally suppressed people with HIV undergoing ART experience both peripheral metabolic damage and CNS dysfunction, as evidenced by the development of neurocognitive impairments [65]. HIV-1 infection induces chronic neuroinflammation, driven by the release of neurotoxic viral proteins such as glycoprotein 120 (gp120), transactivator of transcription (Tat), viral protein R (Vpr), as well as the release of pro-inflammatory cytokines, including tumor necrosis factor-alpha (TNF-α), interleukin-1β (IL-1β), IL-6, IL-8, and IFN-γ [66]. This pro-inflammatory environment disrupts neuronal function by promoting the polarization of microglial M1 pro-inflammatory type and the release of reactive oxygen species [67], leading to synaptic damage, brain energetics disturbance, impaired neuronal integrity, and synaptic loss [68]. Overall, these factors contribute to neurocognitive impairments associated with HIV infection. In a study by Allen et al., gp120 was linked to NCIs observed in people with HIV, possibly by inducing metabolic reprogramming. The study, which investigated cellular processes upstream of mitochondrial functions, demonstrated that gp120 specifically disrupts the glycolysis pathway, increasing the expression of polypyrimidine tract binding protein 1 (PTBP1), which alters the splicing of pyruvate kinase M (PKM) into PKM1 and PKM2. As a result, both the storage of advanced glycation end products (AGEs) and the cleavage of pro-BDNF (brain-derived neurotrophic factor) into mature BDNF are impaired, disrupting synaptic plasticity. However, by stabilizing the tetrameric form of PKM2 with Tepp-46, a potent allosteric PKM2 activator, these effects can be reversed. This suggests that preventing metabolic disruption could help prevent or treat HAND progression [69]. There is also evidence of mitochondrial dysfunction and oxidative stress in the brains of people with HIV [70]. An analysis of postmortem brain samples from 52 patients with HIV revealed a decrease in mitochondrial DNA (mtDNA) content, an increase in the mtDNA common deletion, and a rise in mtDNA point mutations with age [71]. Although ART-naïve people with HIV [70] display mitochondrial dysfunction, long-term ART use has been directly linked with mitochondrial dysfunction in people with HIV [72,73]. It has been demonstrated that NRTIs can differentially influence various mitochondrial functions, leading to cellular stress by inhibiting DNA polymerase gamma (Pol-γ) [74], which is essential for mtDNA replication and maintenance. Among the NRTIs, Efavirenz (EFV), which enhances the release of pro-inflammatory cytokines and causes toxicity [75], has been shown to contribute to cognitive impairments. In a study of 145 patients with HIV, EFV was associated with poorer performance in tests assessing processing speed, verbal fluency, and working memory, compared to patients treated with PIs [76]. In a study by Avdoshina et al., postmortem brain samples from 13 people with HIV and diagnosed with HIV encephalitis (HIVE) were examined. These individuals were identified based on the presence of microglial nodules, astrogliosis, HIV p24-positive cells, and myelin pallor. The study aimed to assess potential changes in mitochondrial size and the integrity of mitochondrial cristae. Using transmission electron microscopy (TEM), the researchers found that, while mitochondrial morphology remained intact in individuals with HIV without HIVE, it exhibited discontinuous and damaged cristae in those with HIVE. Additionally, the mitochondria in HIVE brains were significantly larger, with an increase in size of up to 80% compared to individuals with HIV without HIVE [77]. The study also utilized gp120 transgenic mice, which mimic a HIVE-like condition, to investigate the mechanism by which HIV affects mitochondrial integrity. The findings revealed that gp120 reduced mitochondrial bioactivity by lowering the oxygen consumption rate (OCR), and enhanced the effects of oligomycin in decreasing ATP-linked respiration. Additionally, gp120 reduced mitochondria in neuronal processes, increased mitochondrial size, and impacted mitochondrial movement [77]. Mitochondrial morphology was also altered by the protein Tat, which reduced the mitochondrial membrane potential in rat cortical neurons. Additionally, Tat increased the expression of dynamin-related protein 1 (Drp1), a key regulator of mitochondrial fusion and fission processes, thereby disrupting mitochondrial homeostasis [78]. Another study investigated the sensitivity to changes in mitochondrial integrity in people with HIV undergoing ART. 18 individuals with HIV taking didanosine and/or stavudine, 14 individuals with HIV on zidovudine and lamivudine, 16 individuals with HIV not receiving ART, and 17 HIV− controls were subjected to proton magnetic resonance spectroscopy. In the didanosine and/or stavudine group, there was a significant reduction in frontal white matter mitochondrial *N*-acetylaspartate, compared to HIV− controls. This loss positively correlated with prolonged didanosine and/or stavudine treatment, emphasizing the impact of ART on brain mitochondria depletion and altered cellular respiration [79]. Moreover, a post-mortem analysis of the prefrontal cortex of 20 people who are HIV+ ART-naïve, and 20 people who are HIV+ ART-medicated demonstrated that ART can aggravate white matter pathologies [80]. The HIV+ ART group also increased levels of 2′,3′-Cyclic-nucleotide 3′-phosphodiesterase, a myelin-associated enzyme thought to undergo significant age-associated changes [81], as well as a decrease in myelin basic protein which plays an important role in demyelinating diseases. Recently, some studies have targeted neuroinflammation using adjunctive therapies to ART. For example, the antagonism of the C-C chemokine receptor 5 (CCR5), a receptor implicated in worsening HIV+ neurocognitive impairments [82], reverses these outcomes and ameliorates the cognitive dysfunction in people with HIV. Furthermore, Maraviroc, a CCR5 antagonist, has been shown to decrease levels of neuroinflammation by modulating the activation of monocytes and microglia. Treatment with Maraviroc leads to a significant decrease in pro-inflammatory markers such as soluble CD163 (sCD163) [83], which is a marker for neurocognitive outcomes in children infected with HIV [84] and is linked to neurocognitive impairment in patients with HIV [83]. Insulin resistance contributes to the development of cognitive disorders [85]. Insulin’s ability to regulate various brain functions could serve as another strategy against cognitive impairments, which is why it has recently been studied in relation to dementia and MCI. Intranasal insulin administration in a mouse model of HIV-NCI (EcoHIV-infected mice) produced cerebrospinal fluid insulin levels similar to those seen in patients and, when initiated 23 days or 3 months post-infection, it fully reversed neurocognitive impairment along with associated neuronal and gene expression deficits, the reduction in hippocampal dendritic arborizations, and restored cognitive acuity. Those benefits faded within 24 h of stopping treatment [86]. Intranasal insulin was also tested in people with HIV with cognitive dysfunction. During the Conference on Retroviruses and Opportunistic Infections Boston (CROI, USA, 6–10 March 2021), the first pilot research report NCT03081117 (Intranasal Insulin for the Treatment of HAND) was released. 21 non-diabetic individuals with HIV with mild-to-moderate cognitive deficits were recruited for the randomized, double-blind, placebo-controlled trial. Patients were asked to complete a battery of neuropsychological tests before and after receiving intranasal insulin (INI) (20 IU/day/nare) or a placebo. The INI group demonstrated notable improvements in verbal memory, visual memory, and attention. Another trial NCT03277222 (Intranasal Treatment of HAND) was recently completed, and the results are being analyzed. Figure 2 summarizes the various factors contributing to NCI complications in people living with HIV, which are complex and differ among individuals. These factors include differences in ethnicity, socio-economic status, and nutrition. A deeper understanding of the mechanisms involved in NCIs could help the scientific community develop targeted interventions and, ultimately, improve the care and outcomes for both patients and healthcare providers.

## 4. From HIV to HAND: Exploring the Role of Nutrition

### 4.1. HAND: Classification, Mechanisms and Risk Factors

After the implementation of ART, life expectancy in patients with HIV drastically improved. This therapeutic regimen transformed HIV from a terminal illness into a manageable chronic condition [87]. Nevertheless, due to the chronic nature of the disease, lifelong therapy is necessary. Still, long-term administration of ART has a paradoxical effect, introducing new challenges for people with HIV. In addition to the metabolic dysfunction observed in patients with HIV receiving ART, the neuropsychological manifestations related to HIV, known as HAND, have evolved concomitantly with the widespread adoption of ART. HAND encompasses a spectrum of neurocognitive impairments observed in individuals living with HIV.

The various classifications of HAND were proposed by the United States National Institutes of Health in 2007. The three classifications are known as “*Frascati criteria*” and are classified based on the severity and nature of cognitive impairments in individuals with HIV. **Asymptomatic Neurocognitive Impairment (ANI)** refers to acquired cognitive decline that is measurable in at least two cognitive domains but does not significantly affect daily functioning. **HIV-Associated Mild Neurocognitive Disorder (MND)** describes mild-to-moderate cognitive impairment that causes minor disruptions in daily activities, but does not meet the criteria for delirium or dementia, and cannot be fully attributed to other health conditions. Finally, **HIV-Associated Dementia (HAD)** involves moderate-to-severe cognitive impairment that significantly interferes with daily activities. Similarly to the other categories, it does not meet the criteria for delirium and is not entirely explained by other health conditions [88]. These neurocognitive impairments are closely linked to the ability of HIV to infiltrate the brain early after exposure through a process termed the “trojan horse mechanism” [89,90]. In this mechanism, HIV-infected monocytes cross the blood–brain barrier, leading to the release of viral proteins that can cause neuronal injury. The activation and infection of glial cells by these particles results in a neuroinflammatory state that can further maintain and exacerbate neuronal death [91].

The risk factors related to HIV are multiple, but all revolve mainly around socioeconomic inequities [92]. A vicious cycle exists between poverty and HIV, with each exacerbating the other [93,94]. Food insecurity (FI) further accelerates disease progression, as it is constantly linked to weight loss and wasting [95,96]. The combination of being malnourished, underweight, and having contracted HIV, all worsen immune suppression, leading to the development of AIDS and increasing the risk of opportunistic infections, and, subsequently, death [97,98]. Since diet and nutrition are crucial factors influencing HIV progression, it is also likely that they play a significant role in the development and progression of HAND. Addressing food insecurity, nutrition, and dietary patterns is crucial for understanding HAND development, as well as for mitigating HAND and promoting better cognitive outcomes in individuals with HIV. Moreover, exploring the impact of diet and nutrition on vulnerable and underrepresented subgroups, such as children, the elderly, and individuals with comorbidities, is an important strategy for understanding cognitive decline in populations that are underrepresented in research and that are disproportionately affected by HAND.

### 4.2. Micronutrient Deficiencies and Their Impact on HAND

Micronutrient deficiencies are highly prevalent among people living with HIV, driven not only by the infection itself but also by the lack of access to proper food and diet, particularly in underprivileged communities that face FI [99]. Emerging evidence indicates that deficits in essential vitamins such as A, B6, and D may play a pivotal role in both HIV disease progression and the manifestation of HAND [100,101]. Vitamin A deficiency has been linked to accelerated HIV progression and increased mortality in affected individuals [102]. Moreover, preclinical studies using HIV-1 transgenic rat models subjected to a vitamin A-deficient diet demonstrated impaired motor learning during the rotarod test and decreased exploratory behavior in the open field test [103]. These findings suggest that inadequate vitamin A availability may directly impact cognitive and motor function. Similarly, vitamin B6 (pyridoxine) deficiency has also been linked to neuropsychiatric outcomes in cases of HIV. Patients with HIV and comorbid mood disorders exhibit significantly lower levels of vitamin B6 compared to those without neuropsychiatric symptoms [104,105]. This association is further complicated by coinfection with tuberculosis, where lower B6 levels are accompanied by reduced CD4+ T cell counts, suggesting a potential interplay between micronutrient status, immune function, and mental health [105].

Vitamin D status also appears to influence cognitive outcomes in HIV. Multiple studies have associated low serum vitamin D levels with impairments in memory and fine motor skills, common features of HAND [106]. Notably, a 2021 study by Dong et al. provided the first evidence linking reduced fecal and plasma vitamin D metabolites to neurocognitive impairment in HIV-positive individuals [107]. The study identified a decrease in butyrate-producing bacteria (BPB) and an increase in pathogenic taxa such as *Klebsiella*, implicating gut dysbiosis and altered bile acid metabolism as potential mediators of systemic inflammation and cognitive decline. These microbiota-related mechanisms suggest that vitamin D deficiency may impact HAND through both direct neurological and indirect immunometabolic pathways.

Taken together, these findings underscore the multifaceted role of micronutrients in modulating HIV-related outcomes. Deficiencies in vitamins A, B6, and D are not merely markers of poor nutritional status but likely contributors to the pathophysiology of HAND. Addressing these deficiencies through targeted nutritional interventions may represent a cost-effective strategy to improve both immune function and neurocognitive health in people living with HIV, particularly in socioeconomically disadvantaged populations that remain disproportionately affected.

## 5. Dietary Interventions and Their Influence on HAND

The adoption of diets recognized for their health benefits may enhance immune function, decrease inflammation, improve lipid profiles and cardiovascular health, and reduce the risk of metabolic syndromes in patients with HIV [108,109,110,111]. In the context of HAND, research has shown that the adoption of a ketogenic diet (KD), a low-carbohydrate-intake diet associated with improved cognitive function in several neurological and neurodegenerative disorders [112,113], can enhance cognitive performance in individuals with HIV with HAND [114]. A study by Morrison et al. found that psychomotor speed, executive function and inhibition, processing speed, and verbal memory all improved in patients following a KD [114]. Processing speed and executive function showed the most significant improvements in the experimental group, while the control group exhibited either no improvement or a decrease in the scores across the various neurocognitive tests. The use of KD as a non-pharmacological intervention for managing HAND is promising, as it accounts for the delicate health status of individuals with HIV and the physiological burden of substance use, which is prevalent in this population [114]. Fazeli et al. conducted a cross-sectional study of 86 people with HIV (mean age 56 years) to evaluate how food intervention may influence neurocognitive function in these patients. The results showed that improved diet quality and higher caloric intake were associated with improved cognition in this population [115]. Another study found that a high frequency dietary intake of processed meats, sweet beverages, fish, and whole milk was significantly associated with lower neurocognitive performance in women with HIV (WWH). Notably, results indicated that a higher frequency of vegetable intake was linked to a reduced likelihood of exhibiting an impaired neuropsychological (NP) profile [116]. In contrast, frequent consumption of whole milk was associated with poorer performance in attention, working memory, memory, motor function, and executive function. Among seronegative women from similar socio-demographic backgrounds, only the frequency of sweet beverage intake was associated with impairments in verbal fluency [116]. Moreover, the frequent consumption of processed meats and sweet beverages was associated with reduced motor function. Interestingly, all these associations were found among WWH, but not HIV-seronegative women [116]. Although the rationale for this remains unclear, it is suspected that the metabolic abnormalities associated with HIV infection, exposure to ART, and the possible influence of dietary patterns may contribute to increased susceptibility of WWH to neuropsychological impairments [116]. Nevertheless, more studies are needed to investigate the underlying mechanisms and explore whether dietary interventions could mitigate these impairments.

The association between dietary patterns and neurocognitive and neuropsychological impairments in individuals with HIV must be considered within the broader context of FI. In the general population, FI is associated with cognitive impairment [117,118]. The complexity of FI is that it usually co-occurs with other social hardships and health conditions such as HIV, which may exacerbate their impact on cognitive function [119,120]. Not only do HIV and FI often co-occur, but they may influence one another in a bidirectional manner [121]. Since both have been linked to neurocognitive impairments, it is likely that FI may worsen HAND in individuals with HIV. A study by Hobkirk and colleagues investigated the link between FI and cognitive function in individuals with HIV. Across all participants, both with and without HIV, the presence of FI was associated with cognitive deficits [122]. In addition, patients with HIV who reported FI had significantly higher domain deficit scores in comparison to their food-secure counterparts [122]. This was further corroborated by a significant interaction between HIV infection and FI on several functions, including memory, learning, motor function, and speed of information processing [122]. In addition, Tamargo et al. conducted a 24-month study on individuals with HIV, reporting a significant correlation between FI and cognitive deterioration, depressive symptoms, and cocaine usage. Notably, depression and substance use had no effect on cognitive impairment, as the observed correlation was found in socioeconomically disadvantaged people with limited access to nutrient-dense diets [120]. A personalized mobile health intervention (iSTEP) could improve neurocognitive function in people with HIV by promoting a Mediterranean diet and increasing physical activity [123]. Another study by Rezazadeh et al. investigated the role of nutritional interventions and food policies on HIV-related outcomes in adults. The results indicated that nutritional interventions which included nutrition education, counseling, micronutrient supplementation, and food assistance programs positively impacted the nutritional symptoms and quality of life of people with HIV [124]. Nowadays, various dietary approaches have demonstrated positive effects for lowering the risk of dementia and NCIs in people with HIV. The Dietary Approach to Stop Hypertension (DASH), the Mediterranean diet, and the Mediterranean-DASH Intervention for Neurodegenerative Delay are all effective, but it is crucial to consider all risk factors associated with increased brain vulnerability in HAND [125].

Dietary interventions by themselves are insufficient to lower the overall risk of HAND, and it is crucial to note some of their limitations: the sample size restricts the research’s power, statistics, and generalizability; moreover, many studies fail to evaluate long-term impacts on neurocognitive functioning. Individual differences in metabolism, gut microbiota composition, or genetic predispositions are frequently overlooked by current nutritional therapies, which may not be generally relevant across groups. Lastly, because HAND is complex (including age, immunological activation, viral persistence, chronic inflammation, etc.), dietary modifications might not be sufficient to address all of the contributing aspects. However, by lowering inflammation through different mechanisms, diet intervention may promote overall health and prevent the emergence of HIV-related disorders. Table 2 presents studies conducted on various cohorts of patients living with HIV, who were treated with different dietary regimens in order to assess the impact of diet on cognitive function and the symptomatology of HAND.

## 6. Diet, Nutrition, and HAND: Insights from Vulnerable HIV Subgroups

Studying subgroups within the HIV population, such as children, people with HIV with comorbid infections, and elderly subjects, is essential for understanding HAND and its prevention, as these groups are disproportionately vulnerable to developing neurocognitive impairments. With ART extending life expectancy, the number of elderly people with HIV is slowly increasing within the community [126]. Research on elderly Brazilian people with HIV has demonstrated that patients with low diet quality experience higher levels of anxiety and depression, while those with healthy diets have lower rates of pathological anxiety [127]. This cohort of patients were composed of 112 HIV-positive individuals aged 50 or older, undergoing ART for at least six months, the majority of the sample were male, white, single, with low income and education levels, diagnosed with HIV for over 12 years on average. This study underlined how elderly patients with HIV with poorer diets scored worse in tests related to visual perception and planning, further confirming the association between diet quality, emotional stability, and cognitive function [127]. Similarly, a study by Morrison and colleagues investigated the effects of a KD on elderly patients with HIV with HAND [128]. This study enrolled 20 participants with HIV-associated neurocognitive impairment. Those in the intervention group started a ketogenic diet for 12 weeks, followed by a 6-week return to their usual eating habits. Results demonstrated that the adoption of a KD for 12 weeks significantly enhanced executive function, visuospatial tracking, processing speed, and attention [128].

At the other end of the spectrum, children who are born with HIV have neurodevelopmental impairments that manifest through various cognitive, psychiatric, and behavioral abnormalities [129,130]. A study conducted on 119 HIV-infected Ugandan children between 1 and 6 years of age showed that a low weight-for-age Z score (WAZ), a parameter indicative of undernutrition, is correlated with low cognitive development, as measured by the Mullen Scales of Early Learning [131].

Children exposed to maternal HIV infection in utero or during breastfeeding manifest neurodevelopmental impairments. Interestingly, early-life dietary and nutritional interventions have shown high potential in redirecting neurodevelopmental trajectories and outcomes in these children [132]. Breastfeeding is widely recognized for its positive influence on child neurodevelopment [133]. However, in HIV-exposed children who are particularly vulnerable to neurodevelopmental delays, findings remain inconclusive. While some studies associate breastfeeding with improved cognitive performance in children exposed to HIV in utero compared to those who were not breastfed [134,135], others report no significant link [136]. These inconsistencies may reflect variations in maternal health, ART adherence, or socio-environmental factors. Importantly, the nutritional status of these children plays a critical role, as malnutrition remains prevalent among people living with HIV due to FI [132]. Nutritional interventions, such as macronutrient and multivitamin supplementation, have shown potential in reducing morbidity and mortality in these children [137], with maternal multivitamin use being specifically associated with improved motor development [138]. These findings underscore the need for integrated maternal–child nutritional strategies, including early dietary assessment and supplementation programs, while also calling for larger, well-controlled studies to clarify the neurodevelopmental impact of breastfeeding in HIV-exposed populations. Another study—which involved singleton infants aged between 5 and 7 weeks randomly assigned to receive a daily oral dose of multivitamins or placebo from 6 weeks of age for a duration of 24 months—showed, conversely from other studies, that no association can be found between daily infant multivitamin supplementation and changes in the neurodevelopmental status of children exposed to HIV [139]. Overall, further investigations into the nuanced role of breastfeeding and nutrition are needed to understand their impact on the neurodevelopmental health of children with HIV.

In certain subgroups of people living with HIV, such as those coinfected with both HIV and hepatitis C virus (HCV), elevated coffee intake (ECI) was linked to a decrease in mortality risk by 50% [140]. Since HIV and HCV share similar transmission risks, co-infection is highly likely [141]. In addition, the vulnerability of this cohort to cognitive impairment [142,143] makes it imperative to investigate any potential neuroprotective effects of ECI. A study examining a cohort of 139 HIV/HCV co-infected patients found a positive association between ECI and improvements in neurocognitive performance in executive functioning, verbal fluency, and psychomotor speed [144]. These findings suggest that ECI may help preserve neurocognitive function in people living with HIV and HCV, offering a promising avenue for mitigating cognitive decline in this vulnerable population.

**Figure 3:**
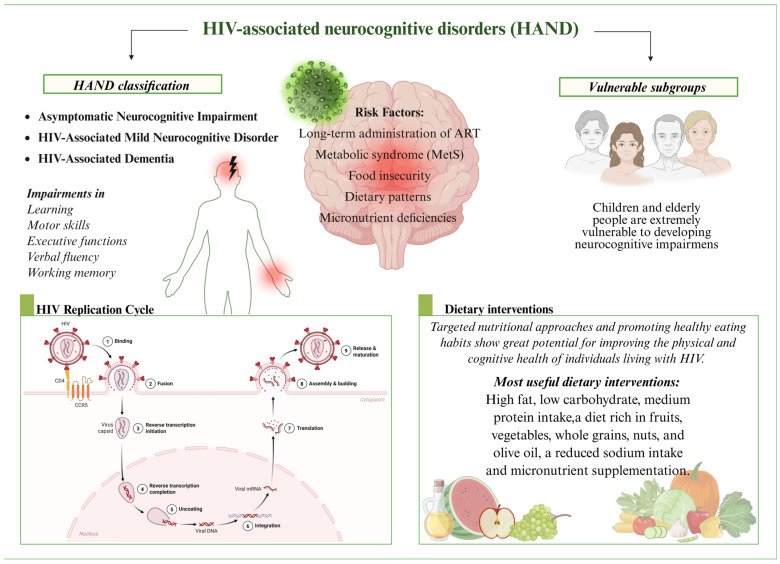
HIV-associated neurocognitive disorders (HANDs), risk factors, and dietary interventions. HAND is classified into three subgroups based on severity: asymptomatic neurocognitive impairment (ANI), HIV-associated mild neurocognitive disorder (MND), and HIV-associated dementia (HAD). These disorders are marked by deficits in learning, motor function, and cognition, including impairments in executive functions, verbal fluency, and working memory. HAND onset involves both the direct effect of HIV infection and viral replication, or it can be attributed to other risk factors that are HIV-related, including long-term antiretroviral therapy (ART), metabolic syndrome (MetS), lack of access to safe and nutritious food (food insecurity), poor dietary patterns, and micronutrient deficiency. Among people living with HIV, children and elderly patients are particularly vulnerable to develop HAND due to developmental and age-related changes that affect the CNS. In addition, prolonged or delayed ART initiation in these subgroups can also increase the risk of HAND. Despite that, they are still underrepresented in HIV research, contributing to inadequate understanding of HAND onset and a lack of optimized treatment interventions. Dietary interventions represent an easy and non-invasive promising adjunctive strategy to improve cognitive outcomes in HIV patients, with commonly used approaches emphasizing high-fat, low-carbohydrate, moderate-protein regimens that are enriched in fruits, vegetables, whole grains, nuts, and olive oil along with a reduction in sodium intake and targeted micronutrient supplementation.

Given the emerging evidence across these vulnerable subgroups, integrating tailored nutritional interventions into routine HIV care may offer a low-cost and accessible strategy to mitigate cognitive decline. Furthermore, the promotion of nutrient supplementation, particularly in early childhood and older age, could support neurocognitive resilience in HIV cases and help prevent the onset of HAND. Figure 3 provides a brief overview of the vulnerable subgroups to HAND and the most commonly used dietary intervention mechanisms.

## 7. Conclusions

The convergence of HIV, metabolic concerns, and HAND emphasizes the complex difficulties that people with HIV currently face. As discussed, metabolic disorders and obesity may be implicated in the impairment of cognitive function in people with HIV due to systemic inflammation. Moreover, impaired glucose metabolism can reduce energy availability for brain cells, which are highly dependent on glucose for optimal function, leading to neuronal damage and disrupted neurogenesis. Additionally, insulin resistance can alter brain insulin signaling, synaptic plasticity, and overall lead to cognitive decline. Dyslipidaemia, which is often observed in individuals with HIV undergoing long-term ART, can increase the risk of developing cardiovascular disease and reduce cerebral blood flow. The latter could deprive the brain of essential nutrients and oxygen, causing brain damage and cognitive dysfunction. Structural changes in the brain, such as reduced gray matter volume or altered white matter integrity, can further negatively affect cognitive health, especially in areas of memory, executive function, and processing speed. Together, these factors create a “vicious cycle,” in which metabolic and cardiovascular issues exacerbate cognitive decline, which in turn makes it more challenging for individuals with HIV to effectively manage these health concerns. Hence, a multifactorial approach, including a well-controlled dietary regimen, may be effective in improving both metabolic and neurocognitive health. In the context of persistent problems associated with HIV, diet, though often overlooked, plays a crucial role in reducing inflammation, improving metabolic function, and supporting brain health. Thus, diet is a key factor that can help control the long-term consequences of HIV. Nutritional strategies, especially those focused on addressing deficiencies and encouraging healthy eating habits, hold considerable promise for enhancing the physical and cognitive well being of individuals living with HIV. Furthermore, subgroups such as children, the elderly, and individuals with comorbidities require targeted dietary strategies due to their increased vulnerability to both HIV and neurocognitive impairments. In order to create comprehensive treatment patterns and achieve the best possible health outcomes that address both the physical and cognitive aspects of the disease, further research is needed to explore the intricate interactions between food, inflammation, and neurocognitive health in people living with HIV. Specifically, more longitudinal studies investigating the effect of various dietary approaches on cognitive trajectories in HAND are needed, especially those that control for confounding factors including comorbidities and food insecurity. Additionally, further studies should aim to provide a clearer link between nutritional factors, neuroinflammation and neurodegeneration in HAND. Overall, these investigations may aid in developing personalized treatment strategies for people with HAND that incorporate both pharmacologic treatment and nutritional and lifestyle approaches tailored to the individual’s socioeconomic status and comorbidities. Additionally, these investigations may aid in identifying biomarkers linking HAND and dietary nutrition, which may enhance the early detection of HAND and inform treatment strategies that can be implemented early on during disease onset.

## Figures and Tables

**Table 1:** The impact of ART therapy on the gut microbiome composition in people living with HIV.

Cohort of HIV Patients	Pharmacological Regimen	Outcome on the Gut Microbiome Composition	Reference
16 viremic patients before and after one year of ART treatment	NNRTI (Zidovudine and Efavirenz)	Reduction in α-diversity	[46]
11 HIV-MetS patients 40 HIV non MetS patients	NRTIs + PIs, 4/11 NRTIs + NNRTIs, 4/11 NRTIs + INSTIs, 2/11 NRTIs + NNRTIs, 18/40 NRTIs + INSTIs, 6/40 NRTIs + PIs, 11/40	Decrease in the abundance of seven genera and seven bacterial species, including some anti-inflammatory bacteria, was observed in the HIV-MetS group	[47]
HIV-MetS patients	INSTI + MetS and PI + MetS	Higher relative abundances of *Bacteroidetes* and *Proteobacteria* in the INSTI + MetS group compared to the PI + MetS; A more pronounced dysbiosis in the INSTI + MetS group characterized by a reduction in α-diversity and an increase in several bacterial genera	[48]

This table summarizes various studies on the gut microbiome composition in HIV-positive individuals under different ART regimens, with or without metabolic syndrome, highlighting the outcomes associated with specific ART therapies in different groups of HIV patients.

**Table 2:** Dietary interventions for HAND with evidence from studies.

Dietary Intervention	Key Features	Impact on Cognition	Proposed Mechanism	Reference	Study Population
Ketogenic Diet (KD)	Emphasizes high fat, low carbohydrate, and medium protein intake	Improves psychomotor speed, executive function and inhibition, processing speed, and verbal memory	Ketone bodies provide an alternative energy source, reduce neuroinflammation, and enhance mitochondrial function	[114]	People with HAND
Improved Diet Quality and Higher Caloric Intake	--	Associated with improved cognition	Supports overall brain function by optimizing nutrient intake and metabolic health	[115]	People with HIV (mean age 56)
Frequent Processed Meat and Sweet Beverage Intake	--	Associated with lower neurocognitive performance	May contribute to metabolic dysfunction, neuroinflammation, and oxidative stress	[116]	Women with HIV
Vegetable-Rich Diet	--	Linked to reduced likelihood of neuropsychological impairment	Provides antioxidants, reduces inflammation, and supports gut microbiome	[116]	Women with HIV
Whole Milk Consumption	--	Linked to poorer performance in attention, working memory, motor function, and executive function	Potential impact on lipid metabolism and insulin resistance affecting brain function	[116]	Women with HIV
Mediterranean Diet (via iSTEP Intervention)	Emphasizes physical activity along with a diet rich in fruits, vegetables, whole grains, nuts, and olive oil	Improved neurocognitive function	Reduces inflammation and supports cardiovascular and metabolic health	[123]	People with HIV
Other Nutritional Interventions	Education, Counseling, Micronutrient Supplementation, Food Assistance	Improved quality of life and nutritional symptoms	Enhances overall nutrition, mitigates malnutrition-related cognitive decline	[124]	Adults with HIV
DASH, Mediterranean, and MIND Diets	Emphasizes a diet rich in fruits, vegetables, whole grains, nuts, and olive oil, as well as a reduced sodium intake	Effective in reducing neurocognitive impairments and dementia risk	Lower inflammation, enhance vascular health, optimize brain metabolism	[125]	People with or without dementia

This table summarizes the findings of different studies conducted across diverse HIV-positive populations (middle-aged adults, women…) investigating different dietary interventions and their effects on HIV-related cognitive deficits. Dietary patterns such as the mediterranean and ketogenic diet have both been associated with improvements in executive function, memory, and overall cognitive performance, while poorer neurocognitive outcomes were linked to frequent intake of processed meats, sweetened beverages, and whole milk. Several mechanisms have been linked to these observed effects, including reduced neuroinflammation, improved mitochondrial and metabolic function, and antioxidant effects. DASH = The Dietary Approach to Stop Hypertension; HAND = HIV-associated neurocognitive disorder; KD = Ketogenic diet; MIND = Mediterranean-DASH Intervention for Neurodegenerative Delay.
